# The Business Outlook

**Published:** 1875-11

**Authors:** 


### THE BUSINESS OUTLOOK.

Everybody is asking, now-a-days, "What is the prospect for business?" and almost everybody answers, "Bad enough." Now, it is not precisely the province of the JOURNAL OF HEALTH to prophecy concerning the future, or to discuss affairs connected with the business world; but as we have observed somewhat closely the condition of affairs since the collapse of values in the fall of 1873, and have some ideas on general business topics, we will offer a few thoughts on the subject.

In the main, things are growing better. The great producing class—the farmers—are far better off to-day than they were last year. All the products of the soil bring good prices, at home and abroad. Multitudes of laborers on railroads and in the mechanic arts have been thrown out of employment by the general business prostration, and this has made farm labor abundant and cheap. Freights have ruled low, and this has enabled all produce to be conveyed to market at moderate cost; and so, as is always the case, the farmer has thrived in spite of the universal prostration of business among other classes.

Economy has been largely practised, sometimes wisely and sometimes unwisely. In the case of the rich, economy has been injudicious, because it has blocked the wheels of the almost paralyzed industries of the land. When times are hard, and factories are closed, and men are being thrown out of employment, the true man, if in the enjoyment of abundant means, comes to the front rank, and circulates his coin, to the relief of his less fortunate fellow-men. This is the course pursued by some of our friends. Dr. Brady, of Williamsburg, L. I., has given employment to a small army of working-men, by the construction of the finest residence for miles around. This is the true charity, after all,—to provide work for those who are too poor even to economise.

The prospect brightens. If those who are poor will continue to practise economy—to live on the proverbial "sixpence a day, and earn it,"—while the rich scatter their money lavishly in building, and in all forms of productive industry, instead of buttoning their pockets and locking their tills,—giving employment and living wages to as many needy and willing workers as possible,—the hard times will be less hard by the end of this winter, and next year will show us activity in all pursuits. Let all who owe money remember that while it is never honest to withhold dollars which are due to another, it is doubly dishonest now that so many are in want; and let us make haste to pay our debts, in order that those whom we pay may in turn pay others.

We do not look for secure times in trade, until our currency is on a level with the coin of the world. So long as our currency is a football to be kicked about by the speculators and "cornerers" of Wall Street, all values must be changable, unstable, and subject to daily fluctuations. There can be little confidence, because a rise or a decline in gold may wipe out a fortune in a day. The buyer of goods, for gold, in Europe, finds that a decline in the greenback price of gold increases the purchasing power of his neighbor's purse, and destroys his own power to compete for the profits of trade. There is nothing left for him but to fail, and his failure carries down a hundred or a thousand others, and seriously cripples as many more. If the price of gold had risen, this man would have survived, become rich, probably, but other thousands would have suffered from a rise in the cost of his commodities. So a fluctuating currency inevitably does infinite harm with every fluctuation; and its greatest evil is its power to destroy confidence.

Next year better times will come, unless the efforts of the glorious army of producers signally fail. With good crops, with a large influx of visitors to help us celebrate our hundredth anniversary, with the presidential canvass and the hopes of better days attending it, we may look forward to a year of activity in most departments of industry, and an autumn of great activity and prosperity. This, at least, is the way it looks to us.

